# Multi-Omics Analyses Reveal Systemic Insights into Maize Vivipary

**DOI:** 10.3390/plants10112437

**Published:** 2021-11-12

**Authors:** Yiru Wang, Junli Zhang, Minghao Sun, Cheng He, Ke Yu, Bing Zhao, Rui Li, Jian Li, Zongying Yang, Xiao Wang, Haiyang Duan, Junjie Fu, Sanzhen Liu, Xuebin Zhang, Jun Zheng

**Affiliations:** 1 Institute of Crop Sciences, Chinese Academy of Agricultural Sciences, Beijing 100081, China; wangyiru91@163.com (Y.W.); sunminghao_yg@yeah.net (M.S.); lr862244377@163.com (R.L.); 13849815737@163.com (J.L.); yangzongying111@yeah.net (Z.Y.); fujunjie@caas.cn (J.F.); 2 State Key Laboratory of Crop Stress Adaptation and Improvement, Henan Joint International Laboratory for Crop Multi-Omics Research, School of Life Sciences, Henan University, Kaifeng 475000, China; zhangjunli0522@163.com (J.Z.); keyu@henu.edu.cn (K.Y.); zhaob@henu.cn (B.Z.); shawnwang2016@126.com (X.W.); 18839046763@163.com (H.D.); 3 Department of Plant Pathology, Kansas State University, Manhattan, KS 66506, USA; ksuhecheng90@gmail.com (C.H.); liu3zhen@ksu.edu (S.L.); 4 Collaborative Innovation Center of Henan Grain Crops, Key Laboratory of Wheat and Maize Crops Science, College of Agronomy, Henan Agricultural University, Zhengzhou 450002, China

**Keywords:** maize, vivipary, seed dormancy, germination, abscisic acid

## Abstract

Maize vivipary, precocious seed germination on the ear, affects yield and seed quality. The application of multi-omics approaches, such as transcriptomics or metabolomics, to classic vivipary mutants can potentially reveal the underlying mechanism. Seven maize vivipary mutants were selected for transcriptomic and metabolomic analyses. A suite of transporters and transcription factors were found to be upregulated in all mutants, indicating that their functions are required during seed germination. Moreover, vivipary mutants exhibited a uniform expression pattern of genes related to abscisic acid (ABA) biosynthesis, gibberellin (GA) biosynthesis, and ABA core signaling. *NCED*4 (*Zm00001d007876*), which is involved in ABA biosynthesis, was markedly downregulated and *GA3ox* (*Zm00001d039634*) was upregulated in all vivipary mutants, indicating antagonism between these two phytohormones. The ABA core signaling components (PYL-ABI1-SnRK2-ABI3) were affected in most of the mutants, but the expression of these genes was not significantly different between the *vp8* mutant and wild-type seeds. Metabolomics analysis integrated with co-expression network analysis identified unique metabolites, their corresponding pathways, and the gene networks affected by each individual mutation. Collectively, our multi-omics analyses characterized the transcriptional and metabolic landscape during vivipary, providing a valuable resource for improving seed quality.

## 1. Introduction

Sophisticated strategies have evolved for seed plants to ensure successful germination. However, precocious seed germination in agriculture, termed preharvest sprouting (PHS) or vivipary, is caused by abscisic acid (ABA) deficiency during the maturation phase and constitutes a severe threat to crop yields. To date, a limited number of vivipary mutants have been reported, such as *phs1*, *phs2*, *phs3*, *phs4*, and *phs8* in rice [1,2]; *not*, *flc* and *sit* in tomato [3,4,5]; and *vp1*/*vp4*, *vp5*, *vp7*, *vp9*, *vp10*/*vp13*, *vp14*, *vp15*, and *y9* in maize [6,7,8,9,10,11,12,13,14], and the feature that these mutants have in common is their decreased ABA content. Genes with different biochemical functions have been identified as being responsible for the vivipary phenomenon. Most of them are involved in hormone signaling, including ABA and gibberellin (GA) signaling [15,16]. ABA and GA antagonistically regulate the seed germination process, with ABA being required for dormancy, whereas GA promotes seed germination [17,18]. The key components of the ABA biosynthesis pathway include phytoene synthase (PSY), phytoene desaturase (PDS), ζ-carotene desaturase (ZDS), zeaxanthin epoxidase (ZEP), 9-cis-epoxycarotenoid dioxygenase (NCED), and abscisic aldehyde oxidase (AAO) [15,18]. The GA biosynthesis rate is mainly determined by reactions catalyzed by GA 20-oxidase (GA20ox) and GA 3-oxidase (GA3ox), whereas GA is inactivated by GA 2-oxidase (GA2ox) [16].

Approximately, 15 viviparous maize mutants have been identified. They have been classified into two groups according to phenotypes: mutants with the color of the endosperm, including *vp1*/*vp4*, *vp6*, *vp8*, *vp10*/*vp13*, *vp14*, and *vp15*; and mutants with altered levels of carotenoids and chlorophyll resulting in the albino phenotype, including *vp2*, *vp5*, *vp7*/*ps1*, *vp9*, *vp12*/*lw2*, *y9*, *w3*, and *rea1* [6]. Maize viviparous mutants have been demonstrated to have defects in ABA biosynthesis or ABA sensing, and multiple maize vivipary-related genes have been cloned. Both the *vp1* and *vp4* genes encode a plant-specific transcription factor involved in ABA signaling that can complement the *Arabidopsis abi3* mutant allele [19,20]. The *vp5*, *vp7*, and *vp9* genes encode enzymes in the carotenoid biosynthetic pathway, and the mutants showed an albino phenotype with reduced ABA levels [8,9,10]. VP14 is homologous to *Arabidopsis* NCED9, catalyzing the first committed step in ABA biosynthesis and cleavage of 9-*cis-*epoxy-carotenoids to form C_25_ apo-aldehydes and xanthoxin, the precursor of ABA biosynthesis in higher plants [12,21]. The final step of ABA biosynthesis is catalyzed by AAO, with molybdenum as a cofactor (MoCo) [22]. Nitrate reductase xanthine dehydrogenase CNX1, CNX2, CNX3, CNX5, CNX6, and CNX7 have been shown to be involved in MoCo biosynthesis in plants [22,23]. In maize, the *vp10* gene encodes ZmCNX1 and the *vp15* gene encodes ZmCNX7. Accordingly, *vp15* and *vp10* mutants are characterized by reduced activity of AAO [11,13]. Apart from the cloned vivipary loci described above, *vp2* has not been cloned; *vp2* mutant accumulates phytoene, the catalyzed product of PDS enzyme, which can regulate the expression of 4-hydroxyphenylpyruvate dioxygenase (HPPD1) [24].

Although multiple genes related to maize vivipary have been identified, the global transcriptional and metabolome impacts from the mutation of viviparous genes have not been systematically investigated. In this study, transcriptomic and metabolomic analyses were performed, using the embryos of seven viviparous mutants, and these revealed a comprehensive molecular network of maize vivipary and potential novel regulators, including transporters, transcription factors, and metabolites during the development of the vivipary phenotype. The present study provides valuable data for breeding maize lines with suppressed vivipary.

## 2. Results

### 2.1. Phenotype of Vivipary Mutants

**Figure 1:**
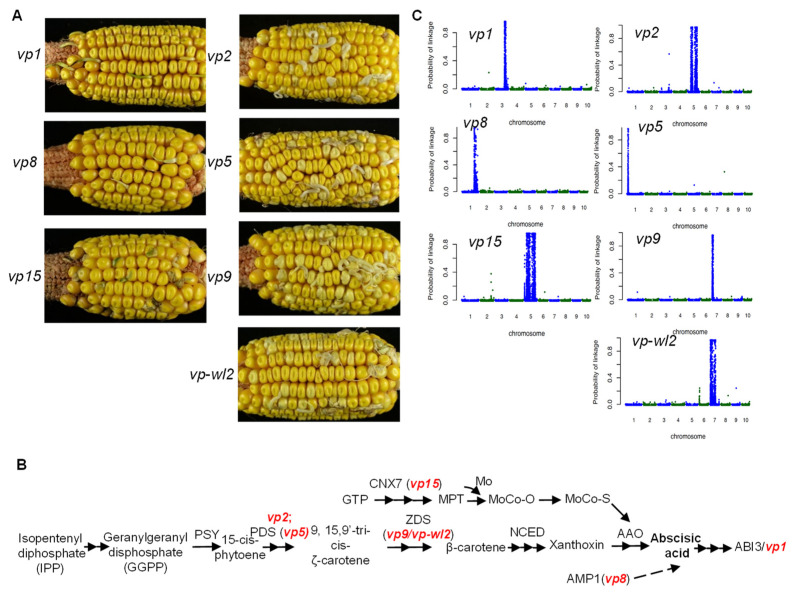
Mapping of each of seven vivipary genes via BSR-Seq. (**A**) Viviparous and normal seeds on heterozygous ears at 60 days after self-pollination of seven viviparous maize mutants *vp1*, *vp2*, *vp5*, *vp8*, *vp9*, *vp-wl2*, and *vp15*. (**B**) The ABA biosynthesis pathway in plants. The red font indicates the position of the mutants in the pathway. (**C**) BSR-Seq results for each viviparous mutant. The physical position of each SNP marker was plotted versus the probability of each SNP marker being in complete linkage disequilibrium with the causal gene (*y*-axis). The interval length of the *x*-axis of each plot was determined by corresponding chromosome length. SNP markers on adjacent chromosomes are distinguished by blue and green dots.

We collected seven viviparous mutants including *vp1*, *vp2*, *vp5*, *vp8*, *vp9*, *vp-wl2* (an allele of the *vp9* locus), and *vp15* for transcriptome sequencing. To reduce the influence of genetic background, each mutant was backcrossed into the B73 inbred line for at least five generations, followed by self-pollination. The kernels of self-pollinated progeny from each line showed a segregating viviparous phenotype (Figure 1A). Viviparous grains in the *vp1*, *vp8*, and *vp15* kernels developed green sprouts, whereas viviparous sprout grains in the *vp2*, *vp5*, *vp9* and *vp-wl2* kernels appeared white or pale yellow (Figure 1A), which was consistent with previous reports [6].

Kernels of each ear with segregating viviparous grains were grouped into mutant seeds (vivipary) or wild-type seeds. Embryos of at least ten mutant seeds or wild-type seeds from the same ear were pooled to represent a pair of mutant and wild-type samples for RNA-Seq. Three biological replicates of mutant and wild-type samples were collected for each viviparous mutant. In total, 42 RNA-Seq libraries were prepared and sequenced. With such an experimental design, BSR-Seq was employed for gene mapping [25]. The mapping locations of the five cloned viviparous genes and the uncloned *vp2* gene were consistent with previous reports, supporting that the correct genetic materials were used (Figure 1C).

### 2.2. Common Biological Processes Affected in Vivipary Mutants

**Figure 2:**
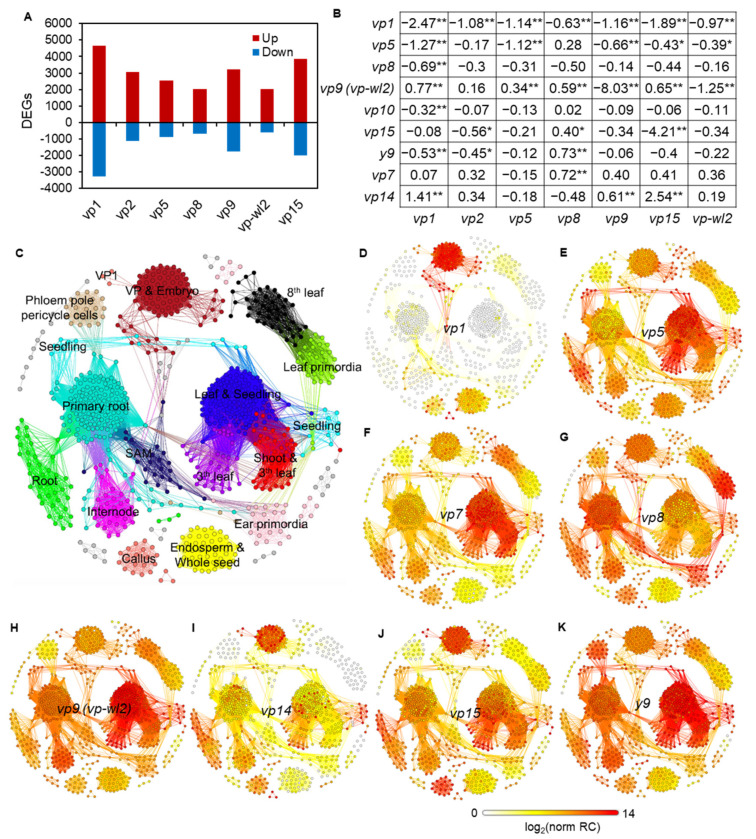
Expression pattern of vivipary genes. (**A**) Number of DEGs in seven comparison groups. Red bars represent upregulated genes in mutants, and blue bars represent downregulated genes in mutants. The DEGs were obtained by comparing the mutant to wild-type (|log_2_ (fold change)| ≥ 1 and FDR < 0.05). (**B**) The reported viviparous genes were significantly expressed in at least one mutant compared with the wild-type. The data are indicated by log_2_ (fold change) of mutants versus wild-types (* *p*-value < 0.05, ** *p*-value < 0.1). (**C**) Tissue-specific sample clusters based on WGCNA analysis: 739 B73 RNA-Seq samples were used to construct the network. Each individual node represented a sample and edges between nodes were determined by the connectivity strength of two samples. “VP1” stands for the *vp1* mutant sample, and “VP & embryo” stands for other vivipary samples except *vp1* and the embryo samples in the 739 additional publicly available data. The different colors represent different modules of B73 RNA-Seq samples. The majority tissues (>90%) of samples in each module were marked on the corresponding module. (**D**–**K**) Expression profile of eight different viviparous genes *vp1*, *vp5*, *vp7*, *vp8*, *vp9* (*vp-wl2*), *vp14*, *vp15*, and *y9* in all samples. The node and edge colors from yellow to red indicate the gene expression levels from low to high. Transparent nodes and edges indicate that the gene is not expressed in this sample. The data was showed by the log_2_ (normalized Read Counts (norm RC)) value.

Analysis of RNA-Seq data produced approximately 24,000 informative genes (genes with at least five reads on average) from each viviparous mutant (Table S1). With at least two-fold expression change and less than the 5% false discovery rate (FDR), we identified 2632 to 7957 significantly differentially expressed genes (DEGs) in the mutants when compared with their corresponding wild-types, with the *vp1* comparison containing the most DEGs (Figure 2A). *vp9* and *vp*-*wl2* are two independent allele mutants that *vp9* contained an 8-bp insertion in the coding region of the ZDS and *vp*-*wl2* mutant had an insertion of Mu9 in the first intron [10]. The different mutant type of *vp9* and *vp*-*wl2* may have different impacts on genes. All known viviparous genes exhibited altered expression levels in at least one comparison (Figure 2B). Interestingly, *vp1* was downregulated in all viviparous mutants, and all other known viviparous genes, except *vp7* and *vp15*, were differentially expressed in the *vp1* mutant (Figure 2B).

Clustering analysis of RNA-Seq samples via weighted gene co-expression network analysis (WGCNA) grouped RNA-Seq samples from this study and 739 additional publicly available RNA-Seq samples of B73 (Table S2) into 16 tissue clusters (Figure 2C). All samples from this study clustered with embryo samples from public samples. The expression of each viviparous gene was displayed on top of this sample network, showing that all viviparous genes were expressed in the embryo (Figure 2D–K). The expression signals of *vp1* (log_2_(norm RC) = 5–13), *vp14* (log_2_(norm RC) = 4–11), and *vp15* (log_2_(norm RC) = 5–11) were the highest in the embryo (Figure 2D,I,J). *vp5* (log_2_(norm RC) = 9–13), *vp7* (log_2_(norm RC) = 6–10), *vp9* (log_2_(norm RC) = 9–13), and *y9* (log_2_(norm RC) = 6–11) had the highest expressions in the seedling stage and likely played a major role in the seedling stage (Figure 2E–K). The expression of *vp8* (log_2_(norm RC) = 5–10) was highest in the leaf primordia and the primary root (Figure 2G), which is consistent with the function of *vp8* in regulating meristem development [26]. To identify functional genes closely associated with vivipary genes, WGCNA was applied to cluster all expressed genes based on all wild-type and vivipary mutant samples. A total of 67 gene modules were obtained, and the four vivipary genes were included in different modules (*vp1* in Module 1, *vp5* in Module 12, *vp9* in Module 26, and *vp15* in Module 15) (Table S3), suggesting that each vivipary gene was involved in different gene networks. Among the 67 gene modules, the *vp1* related module, Module 1, contained the largest number of genes that participate in diverse biological functions, such as transcription factor activity, signal transducer activity, and transporter activity (Table S4). The above analyses suggest that *vp1* may play important roles in maize vivipary.

**Figure 3:**
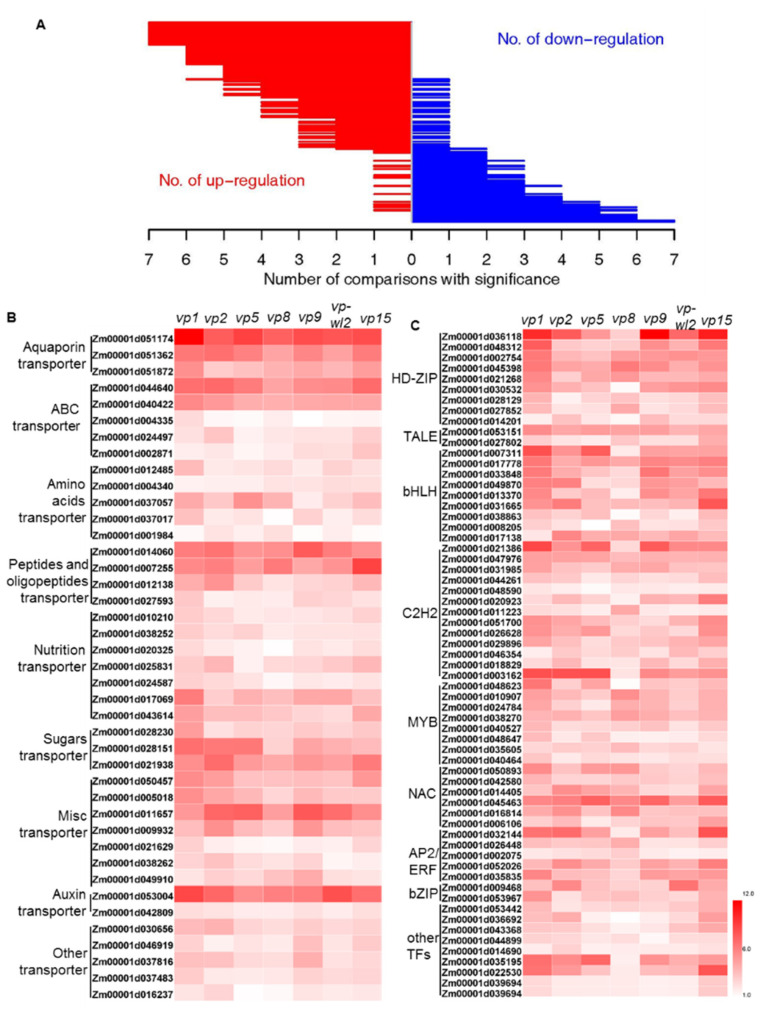
Differentially expression genes (DEGs) between mutants and wild-types of seven vivipary mutants. (**A**) Genes were upregulated or downregulated in the seven comparisons of vivipary mutants versus wild-types. The level of upregulation (red) and downregulation (blue) genes in the vivipary mutants was determined and plotted. The *X*-axis represents the number of comparisons, and the Y-scale represents the level of genes. From top to bottom, the number of upregulated and downregulated genes was used to facilitate sorting. (**B**,**C**) Heatmap analysis of transporters (**B**) and TFs (**C**) showing upregulation in seven vivipary mutants. The DEGs were obtained by comparing the mutant to WT (|log_2_fold change| ≥ 1 and FDR < 0.05).

Among the DEGs, 796 DEGs and 51 DEGs were found to be commonly upregulated and downregulated, respectively, in all vivipary mutants (Figure 3A and Table S5). GO enrichment analysis of the 796 upregulated DEGs in all seven mutants showed that, in biological processes, the metabolic process (GO:0008152), response to stimulus (GO:0050896), and regulation of gene expression (GO:0010468) were enriched; in molecular functions, catalytic activity (GO:0003824), transporter activity (GO:0005215), and oxidoreductase activity (GO:0016491) were significantly enriched. The membrane (GO:0016020) and photosystem (GO:0009521) were significantly enriched in cellular components (Figure S1A). The unique DEGs were identified by hierarchical clustering, and the unique enriched GO terms in each vivipary mutant were also identified (Figure S2), such as cellular amino acid and derivative metabolic processes and the carbohydrate metabolic process being enriched in *vp1*.

### 2.3. Transporters and Transcription Factors (TFs) with Altered Expression in Seven Vivipary Mutants

The genes encoding transporters that are enriched in the common DEGs included aquaporin transporter genes (PIP, Zm00001d051174, and Zm00001d051872; TIP and Zm00001d051362), and nutrition and sugar transporters, including three potassium (K) transporters (Zm00001d010210, Zm00001d038252, and Zm00001d020325), two nitrogen (N) transporters (Zm00001d025831 and Zm00001d024587), one phosphorus (Pi) transporter (Zm00001d017069), one sulfur (S) transporter (Zm00001d043614), and three sugar transporters (Zm00001d028230, Zm00001d028151, Zm00001d021938) (Figure 3B). In addition, five ATP-binding cassette (ABC) transporter family genes, five amino acids transporter genes, four peptides and oligopeptides transporter genes, and two auxin transporter genes were upregulated in the mutants (Figure 3B). These transporters are likely required for water uptake and nutrition relocation during the precocious germination process to sustain the growth of the embryo [27,28,29,30].

Transcription factors (TFs) play pivotal roles in almost all biological processes [31,32]. Among the 796 upregulated DEGs, 62 genes from 15 TF families were identified and enriched in these DEGs (hypergeometric test, *p*-value = 1.93 × 10^−7^). The differentially expressed TF genes were from the families of HD-ZIP (nine genes), bHLH (nine genes), C2H2 (13 genes), MYB (eight genes), NAC (six genes), AP2/ERF (six genes), TALE (two genes), and bZIP (two genes) and were upregulated in all vivipary mutants compared with the wild-type (Figure 3C). HD-ZIP TF (Zm00001d021268) and AP2 TF (Zm00001d009468), which are homologs of ATHB7 and RAV1 in *Arabidopsis*, respectively, were all upregulated in seven vivipary mutants (Figure 3C). ATHB7 and RAV1 act as negative regulators of seed development in *Arabidopsis* by influencing ABA signaling [33,34]. These TFs may act as important transcriptional regulators of seed germination.

To pinpoint the major genes encoding transcription factors and transporters among the common DEGs, we screened the hub genes by exploring the 67 gene modules using intramodular connectivity and gene significance (Table S6). Fourteen transporter genes and thirteen TF genes were identified, including a sugar transporter (Zm00001d028151) in Module 1. Sugar signaling interacts with ABA signaling to regulate seed germination [28] and sugar transporters in vivipary mutants may be important for energy supply or signaling regulation during the seed germination process.

### 2.4. ABA- and GA-Related Genes Were Differentially Expressed in Vivipary Mutants

**Figure 4:**
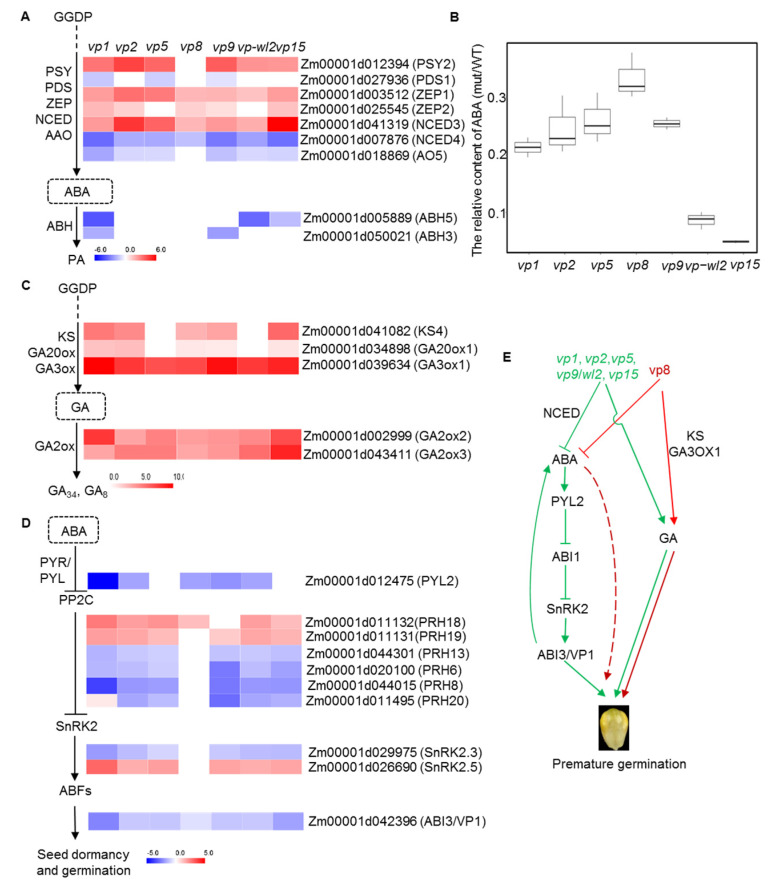
Vivipary mutants affected the biosynthesis and signaling of ABA and GA. (**A**) The expression profiles of ABA biosynthesis-related genes, indicated by log_2_ (fold change) of gene expression in mutants versus wild types, are color-coded. Red boxes represent upregulated genes, and blue boxes represent downregulated genes. (**B**) The relative ABA content of viviparous mutant and wild-type seeds at 30 DAP. Three biological replicates of each sample were conducted. (**C**) The expression profiles of GA biosynthesis-related genes, indicated by log_2_ (fold change) of gene expression in mutants versus wild types, are color-coded. Red boxes represent upregulated genes, and blue boxes represent downregulated genes. (**D**) The expression profiles of ABA signaling-related genes, indicated by log_2_ (fold change) of gene expression in mutants versus wild types, are color-coded. Red boxes represent upregulated genes, and blue boxes represent downregulated genes. (**E**) The pathway of seven vivipary mutants regulated precocious seed germination. Green lines represent the pathway of *vp1*, *vp2*, *vp5*, *vp9*/*wl2*, and *vp15*, and red lines represent the pathway of *vp8*.

As ABA and GA play opposing roles in seed dormancy and germination, we examined the expression of ABA- and GA-related genes in vivipary mutants. In the biosynthetic pathway of carotenoids, precursors of ABA, one *PSY* gene (Zm00001d012394) was upregulated in all mutants except *vp8* (Figure 4A), and one *PDS* gene (Zm00001d027936) was downregulated in mutants of *vp1*, *vp5*, and *vp9* (Figure 4A). However, the expression of two *ZEP* genes (Zm00001d003512 and Zm00001d025545) in the mutants was higher than that in the wild-type (Figure 4A), probably due to the presence of feedback regulation. In the ABA biosynthetic pathway, NCED catalyzes the first committed step [35]. Zm00001d007876 (*NCED4*) was markedly downregulated in all viviparous mutants, but the Zm00001d041319 (*NCED3*) showed the opposite trend (Figure 4A). Zm00001d018869 (*AO5*) was also downregulated in all mutants except *vp8*. The expressions of two abscisic acid 8′-hydroxylase genes (*ABH*) (Zm00001d005889 and Zm00001d050021), which convert ABA into its inactive form, were downregulated in *vp1*, *vp9*, *wl2*, and *vp15* (Figure 4A). Collectively, similar expression patterns of *PSY2*, *ZEPs*, *NCEDs*, and *AO5* were observed in most of the vivipary mutants, indicating that the ABA biosynthetic pathway is subjected to a similar regulation in many vivipary mutants. In addition, we collected embryos from seven viviparous mutants and the wild-type plant at 30 days after pollination (DAP) to quantify the content of ABA. Consistent with previous reports, the ABA content in all mutants was significantly lower than that of the wild type (Figure 4B).

In contrast to ABA, the ent-kaurene synthase gene (KS4, Zm00001d041082), GA20-oxidase gene (GA20ox1, Zm00001d034898), and GA3-oxidase gene (GA3ox1, Zm00001d039634), which are involved in GA biosynthesis, were upregulated in the vivipary mutants (Figure 4C). Notably, the key gene *GA3ox1* in GA biosynthesis was markedly upregulated in all vivipary mutants (Figure 4C). With the presumably enhanced GA biosynthesis, the expression of GA2-oxidase genes (GA2ox2, Zm00001d002999; GA2ox3, Zm00001d043411) in the GA catabolic pathway was also upregulated (Figure 4C).

Mutations in ABA signaling components also alter the degree of seed dormancy [36]. ABA core signaling consists of ABA receptors, group A protein phosphatase type 2Cs (PP2Cs), sucrose nonfermenting 1 (SNF1)-related protein kinases 2 (SnRK2s), and downstream TFs [37]. The ABA receptor (PY2, Zm00001d012475) was downregulated in all mutants except *vp5* and *vp15* mutants (Figure 4D). The key ABA signaling component *PP2C* genes were differentially expressed in most mutants, including two upregulated (Zm00001d011132 and Zm00001d011131) and four downregulated genes (Zm00001d044301, Zm00001d020100, Zm00001d044015, and Zm00001d011495). Zm00001d029975 (SnRK2.3) was downregulated, and Zm00001d026690 (SnRK2.5) were upregulated in most vivipary mutants to regulate the expression of *ABI3*/*VP1* (Figure 4D). However, most of these genes did not exhibit significantly different expressions between the *vp8* mutant and wild-type. These results indicate that the imbalance of ABA and GA as well as altered ABA signaling in vivipary mutants determined the transition from seed dormancy to seed germination, which was conserved between maize and other plants [17]. However, VP8 regulates seed dormancy and germination through other unidentified ABA signaling pathways (Figure 4E). 

### 2.5. Vivipary Mutants Had Distinct Patterns of Metabolite Accumulation

**Figure 5:**
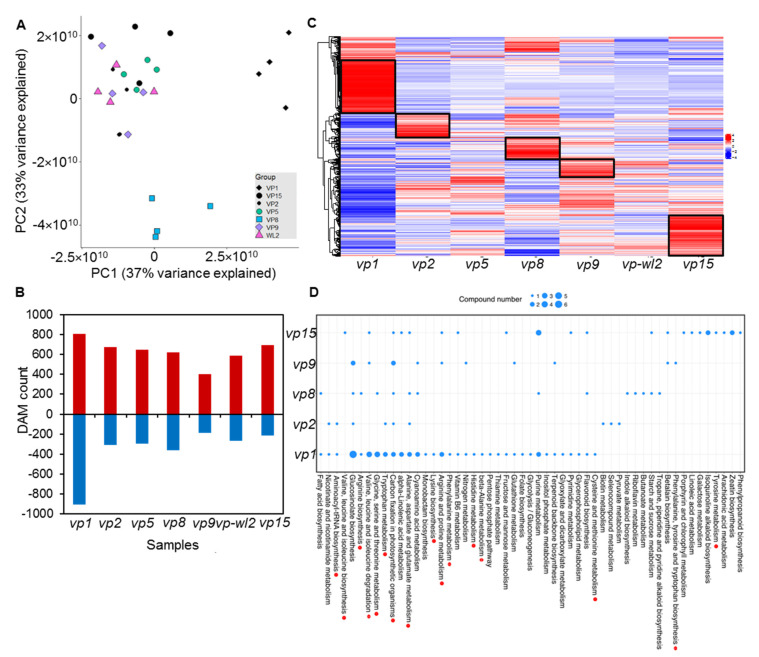
Metabolic profiling of seven vivipary mutants. (**A**) The principal component analysis (PCA) plot demonstrated a distinct separation of seven mutants. (**B**) Different accumulated metabolites (DAMs) in seven comparison groups. Red bars represent increased numbers and blue bars represent decreased numbers of metabolites in the mutants. (**C**) Heatmap of metabolites identified to be significantly discriminatory in the seven comparisons of vivipary mutants versus wild-types. Black boxes represent the specific metabolites in each mutant. (**D**) KEGG analysis of the specific metabolites in seven mutants. Red dots represent amino acid metabolic pathways.

To determine the metabolites highly associated with seed development, we performed global metabolite profiling of vivipary mutants via untargeted metabolome analysis. In total, 3199 metabolites were detected (Table S7). Principle component analysis (PCA) showed that *vp1* and *vp8* mutants were distant from other mutants, indicating distinct metabolite profiles in these two mutants (Figure 5A). Differential analyses of metabolites between each mutant group and the wild-type control identified 590 to 1719 differentially accumulated metabolites (DAMs) from seven comparisons (*p* < 0.05, and at least two fold changes in expression) (Figure 5B). The *vp1* mutant had the highest number of DAMs, in agreement with the highest DEGs from the transcriptomic analysis. We were able to detect reduced the ABA content in all vivipary mutants through untargeted analysis (Table S8), which was highly consistent with the ABA quantification results (Figure 4B). We further correlated the 67 gene modules to all metabolites (Table S9) and found that Module 1 containing *vp1* had the highest mean correlation with metabolites, among which the metabolites with absolute values of Pearson correlation > 0.7 were selected for pathway analysis, revealing that a number of metabolites are involved in amino acid biosynthesis and metabolism (Table S10).

Hierarchal clustering of DAMs from all vivipary mutants specialized in the metabolite profile of each mutant is shown in Figure 5C. We focused on the significantly enriched metabolites in five mutants (indicated by the black boxes in Figure 5C) and performed KEGG enrichment analysis. The number of affected pathways in the *vp1* mutant was the highest in all mutants (Figure 5D), with 14 out of 34 related to amino acid metabolism. Similarly, multiple KEGGs that were enriched in other mutants were identified, and these were classified into aminoacyl-tRNA biosynthesis; valine-leucine biosynthesis; arginine biosynthesis; and alanine, aspartate, and glutamate metabolism pathways, affecting major amino acids including leucine, arginine, tryptophan, lysine, phenylalanine, histidine, L-aspartate. The results correlated well with transcriptional analysis, as five amino acid transporter genes were upregulated in the mutants (Figure 3B).

We were able to identify common and unique enriched KEGG among vivipary mutants (Figure 5D). The metabolite pathways of alanine, aspartate, and glutamate metabolism were commonly affected in all vivipary mutants. “Monobactam biosynthesis”, “lysine biosynthesis”, “phenylalanine metabolism”, “histidine metabolism”, “beta-alanine metabolism”, “pentose phosphate pathway”, “fructose and mannose metabolism”, “glycolysis/gluconeogenesis”, “folate biosynthesis”, “inositol phosphate metabolism”, “terpenoid backbone biosynthesis”, “pyrimidine metabolism”, and “flavonoid biosynthesis” were enriched only in *vp1*. In addition, the “selenocompound metabolism”, “biotin metabolism”, and “pyruvate metabolism” pathways were enriched only in *vp2*. The metabolites in the “indole alkaloid biosynthesis”; “tropane, piperidine, and pyridine alkaloid biosynthesis”; “riboflavin metabolism”; and “starch and sucrose metabolism” pathways were accumulated only in *vp8*. “Porphyrin and chlorophyll metabolism” were enriched only in *vp9.* The metabolites in “caline, leucine and isoleucine degradation”, “betalain biosynthesis”, “linoleic acid metabolism”, “isoquinoline alkaloid biosynthesis”, “galactose metabolism”, “arachidonic acid metabolism”, “tyrosine metabolism”, “phenylpropanoid biosynthesis”, and “zeatin biosynthesis” were accumulated only in *vp15*. Notably, the “purine metabolism” pathway was affected in both *vp1* and *vp15* mutants, and purine metabolism can directly affect ABA biosynthesis [38]. The results indicated that mutations in vivipary mutants have distinct effects on different metabolic pathways.

## 3. Discussion

In the present study, we performed integrated multi-omics analysis using seven maize viviparous mutants and revealed the transcriptional and metabolic landscape of vivipary in maize. We found that the activity of transporters of metabolites including aquaporin, ABC, amino acids, peptides and oligopeptides, nutrition, and sugars was markedly altered during vivipary (Figure 3B). Amino acid and peptide transporters are essential for resource allocation during plant growth and development, especially during seed germination [39,40,41]. AAP8 plays an important role in the uptake of amino acids in embryos and endosperms in *Arabidopsis* [40], and we identified that, among the five amino acid transporter genes, Zm00001d001984, which is a homolog of AtAAP8, was upregulated in all vivipary mutants (Figure 3B). In addition, we detected an overrepresentation of KEGG pathways related to the metabolism of amino acids in five vivipary mutants (Figure 5D), confirming active metabolism and transport of amino acids during vivipary. Meanwhile, metabolites in the “alanine, aspartate, and glutamate metabolism” KEGG pathways were commonly affected in all vivipary mutants (Figure 5D). Aspartate and glutamate were increased during seed germination, which could provide the necessary energy for this high energy-consuming process [42,43,44,45]. Together, transcriptomic and metabolomic analyses indicated that the reallocation of amino acids might play an important role in the precocious seed germination of vivipary mutants.

In addition to amino acid transporters, genes encoding four peptide transporters (PTRs), five ABC transporters, and three sugar transporters, were found to be upregulated in our mutants, suggesting that they may play a role in seed germination (Figure 3B). PTR2 in *Arabidopsis* is negatively regulated by ABI4 and plays a key role in water uptake by seeds, ensuring that imbibed seeds proceed to germination [27], and ZmPTR1, VfPTR1, VfPTR2, and HvPTR1 are for seed germination in maize, faba beans, and barley [46,47]. Accordingly, three aquaporin transporters were upregulated in the vivipary mutants (Figure 3B), likely facilitating water uptake during seed germination. Several members of the ABC transporter have been reported as free ABA transporters [48]. ABCG20 is necessary for normal seed dormancy in *Arabidopsis*, and MtABCG20 is an ABA exporter that influences seed germination of *Medicago truncatula* [49,50]. The upregulation of ABC transporters in vivipary mutants probably facilitates the export of ABA from the embryo. Sugars are a major energy resource in biological processes, and sugar signaling interacts with ABA signaling to regulate seed germination [28]. In crops, the rice sugar transporter OsSUT1 [51,52] and maize ZmSUT1 [29] are highly expressed in germinating seeds, and the suppression of OsSUT1 resulted in retarded germination [30]. The increased expression of sugar transporters in vivipary mutants may be important for energy supply or signaling regulation during seed germination.

TFs play pivotal roles in seed dormancy and germination. We identified 62 TF-encoding genes that were upregulated in all vivipary mutants. Among these, C2H2, HD-ZIP, bHLH, and MYB were the most abundant (Figure 3C). Previous studies have indicated that at least three class I HD-ZIPs (AtHB6, AtHB7, and AtHB12) participate in ABA signaling as part of a negative feedback loop containing the ABI and ABA receptors [33,53,54]. Nine HD-ZIP TFs, including Zm00001d021268, which is a homolog of ATHB7, were upregulated in all mutants (Figure 3C). The AP2 TF RAV1, as a negative regulator of seed development in *Arabidopsis*, directly binds to the promoters of ABI3 and ABI4, leading to the perturbation of ABA signaling [34,55]. Zm00001d009468, a homolog of *RAV1*, was also upregulated in vivipary mutants, whereas the expression of *VP1*, a homolog of *ABI3*, was reduced (Figure 3C), indicating a similar function of maize *RAV1* in the suppression of ABI3. Meanwhile, many bHLH and MYB TFs were found to be specifically ABA-repressed during seed dormancy [56]. A group of MADS-box, MYB, NAC, and WRKY genes have also been implicated in seed dormancy regulation [57]. We identified a number of bHLH, MYB, and NAC TFs that were induced in seven vivipary mutants, indicating that these genes are likely released from ABA repression during seed germination.

Seed dormancy and germination are regulated by a wide range of plant hormones, including ABA, GA, ethylene, and brassinosteroid, of which ABA and GA are the primary factors for seed dormancy and germination [15,58]. We confirmed that all seven vivipary mutants accumulated significantly less ABA (Figure 4B and Table S8). Most enzymes involved in ABA biosynthesis were identified (Figure 1B). PSY catalyzes the first committed step in carotenogenesis [59]. We found that *PSY* was upregulated in all the selected mutants, except for *vp8* (Figure 4A). This is likely due to feedback regulation, as most of the *VP* genes are located downstream of *PSY*. Xanthophyll cleavage by NCED is the first committed step in ABA biosynthesis and is rate-limiting [15,35]. *NCED3* was upregulated while *NCED4* was downregulated in all mutants. *NCED4* (Zm00001d007876) is a homolog of *AtNCED9* and *VP14*. Loss of function of *AtNCED9* or *VP14* in *Arabidopsis* or maize leads to decreased endogenous ABA content and thus reduced seed dormancy [12,60]. Therefore, the reduced ABA content in all the mutants is likely a result of downregulated *NCED4* expression, indicating a key role of NCED4 but not NCED3 in ABA biosynthesis and seed dormancy maintenance in maize. ABA controls seed dormancy and germination via a complex signaling network. The core components from ABA perception to ABA-regulated gene expression (PYR/PYL/RCAR-ABI1/2-SnRK2s-ABFs/AREBs) have been reported [61]. As expected, the positive seed dormancy genes (one ABA receptor: Zm00001d012475; one SnRK2: Zm00001d029975; and ABI3/VP1: Zm00001d042396) were all downregulated in all mutants except *vp8*, whereas the negative seed dormancy genes (two PP2Cs: Zm00001d011131 and Zm00001d011132) were upregulated in vivipary mutants. Zm00001d011131, a homology of ABI1, was upregulated in all mutants except *vp8*. The metabolite PCA also showed that *vp8* was separated from other mutants, indicating the distinct role of *vp8* in seed dormancy and germination, which may affect another signaling pathway regulating seed dormancy (Figure 4E and Figure 5A). VP1 and ABI3 are key determinants of seed-specific gene expression [20,62]. VP1/ABI3 strongly modifies ABA signaling by regulating of members of *ABI1*/*ABI5*-related gene families [63]. Moreover, transgenic wheat constitutively expressing the *vp1* gene enhanced seed dormancy and PHS tolerance [64]. HAI2 (a PP2C) negatively regulates the ABA response, and its mutation leads to a deep dormancy phenotype in *Arabidopsis* [65]. However, Zm00001d020100, Zm00001d044015, and Zm00001d011495, the homologs of *HAI2*/*3*, were downregulated in our vivipary mutants (Figure 4D), indicating the different regulation strategies adopted in different plant species.

The maintenance of seed dormancy by ABA and dormancy release by GA has been observed in many species. Dormancy release largely reflects increased GA synthesis, which is dependent on the increased expression of *GA3ox1* and *GA3ox2* [66]. In seven vivipary mutants, the expression of Zm00001d039634 (*GA3ox1*) was markedly induced (Figure 4C), likely resulting in the accumulation of GA. Overall, seven vivipary mutants affected seed dormancy and germination by inhibiting ABA synthesis and by promoting GA synthesis (Figure 4E).

In conclusion, our multi-omics analysis of maize vivipary mutants supported the proposition that ABA and GA biosynthesis and signaling are the causes of vivipary. We also revealed common and unique biological processes, transcriptional regulators, and metabolic pathways that are possible regulators of the viviparous phenotype. The data presented in this study can be utilized for the genetic improvement of maize against vivipary.

## 4. Materials and Methods 

### 4.1. Plant Materials

The reference mutant alleles of each gene (*vp1*, *vp2*, *vp5*, *vp8*, *vp9*, *vp-wl2*, and *vp15*) were introduced to the inbred line B73 via at least five backcrosses. Self-pollination of heterozygous individuals from the last backcrossing seeds generated segregating families. We selected normal seeds as the wild-type and viviparous seeds as the mutant at 30 DAP.

### 4.2. RNA Extraction and Sequencing

For RNA-Seq experiments, wild-type and mutant embryos from each family, collected as described above, were separately pooled and stored at −80 °C for RNA extraction. At least 10 embryos per pool were collected for each sample, and 3 biological replicates of each sample were used for RNA-Seq.

Total RNA was extracted using the RNeasy Plant Mini Kit (Qiagen). RNA concentration and purity were determined using a Nanodrop ND-2000 spectrophotometer. cDNA libraries were sequenced with a read length of 150 bp (paired-end) using an Illumina HiSeq 2000 System at Annoroad Genomics (Beijing, China). Sequencing libraries were generated using the VAHTS Universal V6 RNA-seq Library Prep Kit for Illumina^®^ (NR604-01/02) following the manufacturer’s recommendations, and index codes were added to attribute sequences to each sample. mRNAs were isolated using oligo(dT) magnetic beads (Illumina). RNA fragmentation, cDNA synthesis, and PCR amplification were performed, and the final cDNA library was obtained by PCR enrichment. To reduce technical variation during sequencing, all six samples associated with a given viviparous gene were sequenced in the same lane. Quality checks were performed using the FastQC software [67]. Raw reads were trimmed to remove adaptors and low-quality base pairs using Trimmomatic (v3.6) [68]. The remaining clean reads were then mapped to the maize B73 reference genome (AGPv4) using STAR [69]. Raw read counts were used to calculate FPKM values. Genes with expression |log_2_ (fold change)| ≥1 and FDR < 0.05 were considered DEGs.

### 4.3. BSR-Seq Mapping

Genomic locations of viviparous genes were determined using bulked segregant RNA sequencing (BSR-Seq) [25].

### 4.4. Gene Ontology Analysis

Gene ontology (GO) analysis of DEGs was performed using the online tool AgriGo (http://bioinfo.cau.edu.cn/agriGO, accessed on 10 August 2021) [70]. GO terms with adjusted *p*-values smaller than 0.05 were considered significantly enriched terms.

### 4.5. Analysis of ABA Content

ABA was quantified using ultra-high-performance liquid chromatography–tandem mass spectrometry (UPLC–MS/MS). The samples were analyzed using a Xevo TQ-XS system (Waters, Milford, MA, USA) equipped with an ESI ion source. Chromatographic separation was conducted using an ACQUITY UPLC HSS T3 column (2.1 × 100 mm, 1.8 µm) maintained at 40 °C. The autosampler temperature was set to 4 °C, and the sample injection volume was 10 μL. The MS data were collected in the negative ion mode using multiple reaction monitoring (MRM) mode. Precursor and fragment ions were ABA (*m*/*z* 263.16–153.01) and d_6_-ABA (*m*/*z* 269.20–159.10). Data analysis was performed using a spectrometer software (Masslynx v.4.2).

### 4.6. WGCNA Network Construction and Module Identification

B73 RNA-Seq data were downloaded from the NCBI Sequence Read Archive (SRA) (Table S2), which was generated from multiple tissues, including but not limited to seedlings, leaves, crown roots, tap roots, whole roots, shoots, seeds, steles, shoots, stems, pollen, ears, and tassels. Raw reads were trimmed to remove adaptors and low-quality base pairs using Trimmomatic (v3.6) [68]. Clean reads were aligned to B73Ref4 using STAR [69]. The read counts for each gene were normalized to library size. Normalized data were used to construct the tissue network using the R package WGCNA (version 1.66) [71]. WGCNA was performed to cluster 739 B73 samples and 42 vivipary samples with the parameters minModuleSize = 20 and soft-thresholding power = 12. If >90% of the samples in one module belonged to the same tissue, then the module was annotated as this tissue. The connectivity between the two samples ranged from 0 to 1. Higher scores indicate stronger connectivity strength and are shown as deeper color edges in the network (Table S11). Gephi software (version 0.9.2) was used to visualize tissue networks with the module and connectivity information from the WGCNA results. The expression of each vivipary gene was converted to a shade of red and marked in the tissue network.

Samples of the wild-type and vivipary mutants were used to construct the co-expression network using the R package WGCNA. The gene significance (GS) quantifying associations of individual genes with the vivipary phenotype and the module membership (MM), which acted as the correlation between the module Eigen genes and the gene expression profiles, were calculated. Genes with a GS > 0.75 and an MM > 0.85 were considered major genes.

### 4.7. Metabolomic Analysis

Wild-type and mutant embryos from seven viviparous materials were collected for metabolomic analysis. The powder of each sample was extracted overnight using 80% HPLC-grade methanol containing 1 μM chrysin as the internal standard. The ratio of the fresh weight to the volume of extraction solution was kept at 0.1 g/mL. The undissolved sample residues were precipitated by centrifugation at 13,000× *g* rpm for 30 min at 4 °C. The clear supernatants were loaded into injection vials for UHPLC–MS/MS.

For the UHPLC–MS/MS assay, the vanquish-flex UHPLC system was coupled to a Q Exactive Plus mass spectrometer (Thermo Fisher Scientific, Bremen, Germany) for metabolite separation and detection. Following LC–MS analysis, raw data were collected and processed using Compound Discoverer 3.2 (Thermo Fisher Scientific) with the metabolite databases mzCloud, mzVault, Masslist, and Chemspider. A principal component analysis (PCA) was performed directly. Heatmap and KEGG analyses of metabolites were performed using R software (version 3.6.3).

## Data Availability

The RNA-seq data in this study were deposited in the NCBI sequence read archive (SRA) with the accession number PRJNA773210. The results of transcript quantification (raw reads count) and metabolites are provided as Supplementary Materials.

## Supplementary Materials

The following are available online at https://www.mdpi.com/article/10.3390/plants10112437/s1. Supplementary Figure S1: GO category analysis of DEGs in seven vivipary mutants. Supplementary Figure S2: Gene ontology (GO) category analysis of the specific genes in vivipary mutants. Supplementary Table S1: The summary of RNA-Seq. Supplementary Table S2: Information on the 739 B73 RNA-Seq samples. Supplementary Table S3: Sixty-seven modules obtained following WGCNA analysis. Supplementary Table S4: GO categories analysis of the genes in Module 1. Supplementary Table S5: The commonly up- and downregulated genes in seven vivipary mutants. Supplementary Table S6: The module membership and gene significance of transporters and TFs. Supplementary Table S7: The summary of metabolites. Supplementary Table S8: The relative level of metabolite abscisic acid in vivipary mutants. Supplementary Table S9: The correlation of gene modules with metabolites. Supplementary Table S10: KEGG analysis of the metabolites that had high mean correlation with Module 1. Supplementary Table S11: The connectivity of the tissue samples in the network
