# Supplementary material for: Multi-Omics Analyses Reveal Systemic Insights into Maize Vivipary
Source: Plants (Basel). 2021 Nov 12;10(11):2437. doi: 10.3390/plants10112437 (PMC8618366; doi:10.3390/plants10112437)
Supplement: Supplementary file 1 [file plants-10-02437-s001.zip › plants-1414738-supplementary.pdf]

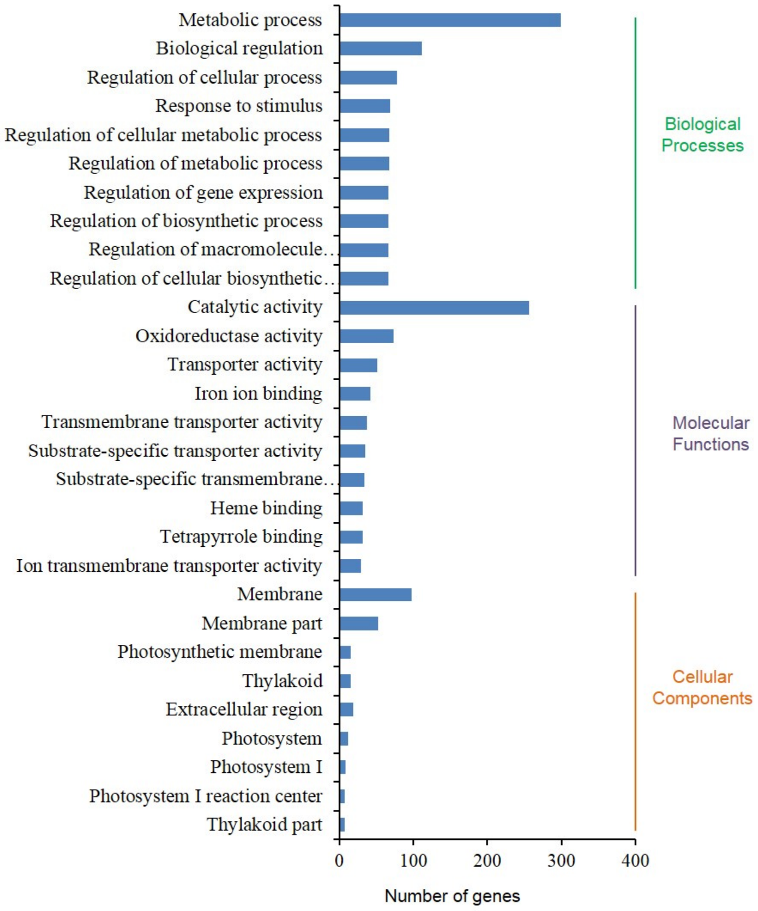

Figure S1. Gene ontology (GO) category analysis of DEGs in seven vivipary mutants. Significant gene GO terms ($q < 0.05$) from the GO enrichment test are listed.

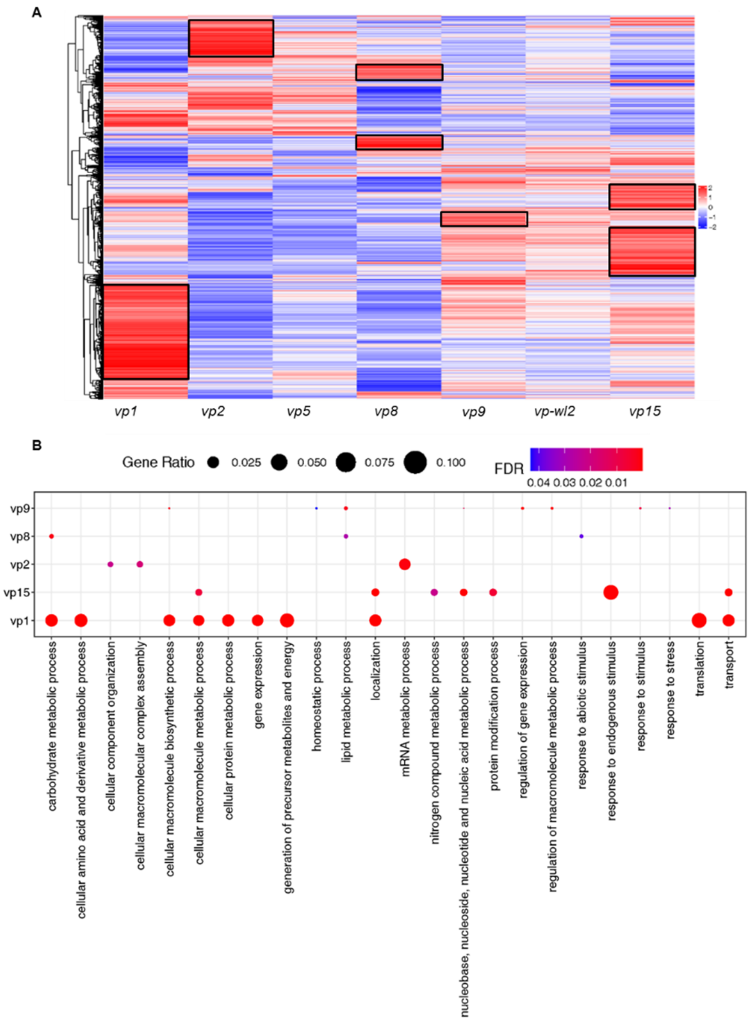

Figure S2. Gene ontology (GO) category analysis of the specific genes in vivipary mutants. (A) Heatmap of genes identified as significantly discriminatory in the seven comparisons of vivipary mutants versus wild-types. Black boxes represent the specific genes in each mutant. (B) KEGG analysis of the specific genes in seven mutants.
